# Severe Hypotension, Hypoxia, and Subcutaneous Erythema Induced by Indigo Carmine Administration during Open Prostatectomy

**DOI:** 10.1155/2016/5237387

**Published:** 2016-08-16

**Authors:** Koichiro Nandate, Bryan B. Voelzke

**Affiliations:** ^1^Department of Anesthesiology and Pain Medicine, Harborview Medical Center, University of Washington, 325 Ninth Avenue, P.O. Box 359724, Seattle, WA 98104-2499, USA; ^2^Department of Urology, Harborview Medical Center, University of Washington, 325 Ninth Avenue, P.O. Box 359868, Seattle, WA 98104-2499, USA

## Abstract

Indigo carmine (also known as* 5,5*′-*indigodisulfonic acid sodium salt* or* indigotine*) is a blue dye that is administered intravenously to examine the urinary tract and usually is biologically safe and inert. Indigo carmine rarely may cause adverse reactions. We treated a 66-year-old man who had general anesthesia and radical retropubic prostatectomy for prostate cancer. He had a previous history of allergy to bee sting with nausea, vomiting, and dizziness. Within 1 minute after injection of indigo carmine for evaluation of the ureters, the patient developed hypotension to 40 mmHg, severe hypoxia (the value of SpO2 (peripheral capillary oxygen saturation) was 75% on 40% inspired oxygen concentration), poor air movement and bilateral diffuse wheezing on auscultation, and marked subcutaneous erythema at the upper extremities. After treatment with 100% oxygen, epinephrine (total, 1.5 mg), hydrocortisone (100 mg), diphenhydramine (50 mg), albuterol nebulizer (0.083%), and continuous infusion of epinephrine (0.15 *μ*g/kg/min), the vital signs became stable, and he recovered completely. In summary, indigo carmine rarely may cause life-threatening anaphylactic or anaphylactoid reaction that may necessitate rapid treatment to stabilize cardiovascular, hemodynamic, and pulmonary function.

## 1. Introduction

Indigo carmine (also known as* 5*,*5*′-*indigodisulfonic acid sodium salt* or* indigotine*) is a blue dye that has been used since the early nineteenth century to localize the ureteral orifice and identify severed ureters and urinary fistulas [1]. Although usually safe for clinical use, indigo carmine occasionally may induce severe hypertension and bradycardia, possibly by stimulation of alpha receptors. Hypertension after indigo carmine injection may occur possibly because of the common chemical structure between indigo carmine and the neurotransmitter serotonin (5-hydroxytryptamine). Serotonin directly causes vasoconstriction and positive inotropic effects mediated through the alpha-adrenergic receptor. Therefore, administration of indigo carmine may increase total peripheral resistance, resulting in elevated blood pressure followed by a bradycardic reflex [2–4]. In addition, catastrophic adverse reactions after intravenous administration of indigo carmine have been reported, including critical hypotension and anaphylactic reaction, but the pathologic mechanism is unknown [5–7].

We treated a patient who had anaphylactic reaction after an intravenous injection of indigo carmine during radical retropubic prostatectomy. This reaction is not new and has been already reported. However, it is worthwhile being reported again to warn anesthesiologists, urologists, and gynecologists of life-threatening reaction by indigo carmine.

## 2. Case Presentation

A 66-year-old man presented with prostate cancer. He did not have either any major medical problems including cardiovascular or respiratory diseases, history of surgical procedure, or exposure of indigo carmine. He had a history of allergic reaction to bee stings associated with nausea, vomiting, and dizziness, which had been treated in the emergency department. He was scheduled to undergo elective radical retropubic prostatectomy with pelvic lymph node dissection under general anesthesia.

After induction of general anesthesia with midazolam, fentanyl, and propofol, muscle relaxation was achieved with rocuronium, and the trachea was intubated. An arterial line and 2 large intravenous catheters were placed, according to our routine for patients undergoing radical prostatectomy. No unusual events were noted during general anesthesia induction and preparation for the surgery.

There were no issues during the operation until the surgical team requested the anesthesia team to give 5 ml of indigo carmine (0.8% sodium indigotindisulfonate USP solution, America Regent Company, Shirley, NY, USA) intravenously to ensure that neither ureter was injured during prostatectomy. By this time, estimated surgical blood loss was 1500 mL, and the patient had been given 2 units of red blood cells, 3500 mL crystalloid, and low doses of vasopressors (phenylephrine 0.1 microgram/kg/min) to stabilize the vital signs. Hematocrit was 31%. Within 1 minute after administration of indigo carmine, the vital signs deteriorated suddenly. Systolic blood pressure dropped from 110 to 40 mmHg, but the heart rate remained at 60 beats/min. The patient became hypoxic (oxygen saturation decreased from 99% to 75% on 40% inspired oxygen concentration). Auscultation showed poor air movement and bilateral diffuse wheezing. Marked cutaneous erythema was observed at the upper extremities.

The anesthesia team requested the surgical team to suspend surgery temporarily until the patient became stable. The patient immediately was given 100% oxygen, epinephrine (total, 1.5 mg), hydrocortisone (100 mg), diphenhydramine (50 mg), albuterol nebulizer (0.083%), and continuous infusion of epinephrine (0.15 *μ*g/kg/min), and the vital signs became stable. Emergency transesophageal echocardiography was performed, and there was no evidence of myocardial infarction or pulmonary embolism. The diagnosis of anaphylactic reaction due to indigo carmine was made on the basis of the sudden decrease in blood pressure, respiratory problems, and subcutaneous lesions immediately after the administration of indigo carmine.

After the vital signs were stabilized, the surgery was resumed and completed uneventfully. The patient remained on a continuous infusion of epinephrine (0.1 *μ*g/kg/min) and was transferred to the surgical critical care unit for close monitoring without emerging from general anesthesia. The patient was released from the critical care unit to the ward after 48 hours and discharged from the hospital on postoperative day 7 without any further complications. During the patient's stay in the hospital, we seriously considered investigating the serum activity of tryptase, histamine, and immunoglobulin E but could not achieve the patient and the family agreement.

## 3. Discussion

The present patient had an anaphylactic reaction, manifested by hypotension, hypoxia, bilateral wheezing, and subcutaneous erythema, after intravenous injection of indigo carmine.

In a previous report of 4 patients who were treated within 6 weeks for severe hypotension, none of the patients had a history of allergy, previous exposure to indigo carmine, or anaphylactic signs such as cutaneous erythema, laryngeal edema, or bronchospasm; the authors were unable to link the hypotensive reaction to an anaphylactic reaction and warned about the possibility of drug lot impurity [5]. We examined the lot impurity of indigo carmine which we gave to the patient but it was far before the expired date. In two other previous case reports, the marked hypotensive response noted immediately after intravenous administration of indigo carmine was due to anaphylactic reaction [6, 7]. In one of these case reports, the patient had the complete spectrum of anaphylaxis but did not have a history of allergies or previous exposure of indigo carmine, and the authors suggested that indigo carmine directly may have triggered histamine release, consistent with an anaphylactoid reaction associated with severe hypotension, bronchospasm, and urticaria [6]. In the other case report, the patient did not have a previous history of allergy or skin symptoms, but he had severe hypotension and hypoxia, with wheezing that progressed to cardiac arrest [7].

The present patient had a history of bee sting allergy, and he developed hypotension, wheezing, and subcutaneous erythema within several minutes after the administration of indigo carmine. It may be difficult to prove whether he had a life-threatening anaphylactic reaction in response to indigo carmine. The only proven method to evaluate the patient's allergic response to indigo carmine is skin testing. We seriously considered performing the skin testing, but the patient refused. Therefore, anaphylactic reaction usually may be diagnosed on the basis of a previous history of allergy and the clinical presentation. The clinical condition of the present patient satisfied the clinical criteria for a life-threatening reaction due to anaphylactic response by the World Allergy Organization [8].

In summary, indigo carmine may be used in routine clinical practice in urology or gynecology because of the safety profile of the dye compared with methylene blue [9]. However, physicians should be aware that indigo carmine rarely may cause a major life-threatening anaphylactic reaction.
